# Thyroid storm during the recovery phase after non-thyroid surgery in a hyperthyroid patient: a case report and literature review

**DOI:** 10.3389/fsurg.2025.1633314

**Published:** 2025-07-14

**Authors:** Jiyu Zeng, Ting Yang, Li Wu

**Affiliations:** ^1^ Department of Anesthesiology, West China Hospital of Sichuan University Ziyang Hospital, Ziyang, China; ^2^ Department of Medical Education, West China Hospital of Sichuan University Ziyang Hospital, Ziyang, China

**Keywords:** thyroid storm, perioperative management, hyperthyroidism, non-thyroid surgery, case report

## Abstract

**Background:**

Thyroid storm is a life-threatening endocrine emergency characterized by an acute exacerbation of thyrotoxicosis, often triggered by stressors such as surgery or infection, with a mortality rate of 8%–25%. Although the risk is well-documented in thyroid surgeries, perioperative thyroid storm following non-thyroid procedures is exceedingly rare, posing diagnostic and therapeutic challenges. This case report and literature review aim to highlight the clinical features and management strategies for perioperative thyroid storm in non-thyroid surgical patients through a case analysis and literature review.

**Case presentation:**

A 53-year-old Chinese male with a 20-year history of poorly controlled hyperthyroidism (irregular medication adherence) underwent closed reduction and intramedullary nailing for a right femoral fracture. Preoperative evaluation revealed mildly elevated free triiodothyronine (FT3: 6.87 pmol/L) and profoundly suppressed thyroid-stimulating hormone (TSH: <0.01 mIU/L). Antithyroid medication was omitted on the day of surgery. Following surgery and transfer to the recovery room, the patient demonstrated delayed emergence from anesthesia, with a Burch-Wartofsky score of 45 and persistent tachycardia (heart rate 144 bpm), meeting Grade 1 thyroid storm criteria per Japan Thyroid Association guidelines, indicating a definitive thyroid storm. After about one hour, the patient was diagnosed with thyroid crisis. Intravenous hydrocortisone (100 mg) and continuous esmolol infusion were promptly initiated, leading to gradual heart rate stabilization at 120 bpm. Approximately 20 minutes later, the patient regained full consciousness and met criteria for discharge from the recovery room. The patient was discharged on postoperative day 10 without complications.

**Conclusions:**

This case underscores that non-thyroid surgery can precipitate thyroid storm in hyperthyroid patients, even with atypical presentations (e.g., absence of hyperpyrexia). Early recognition relies on vigilance toward tachycardia and altered mental status. Perioperative management should emphasize: (1) rigorous preoperative optimization of thyroid function to achieve euthyroidism; (2) vigilant postoperative monitoring for early signs of thyroid storm; and 3) prompt diagnosis using the Burch-Wartofsky scale and guideline-based criteria, followed by combined therapy with beta-blockers, corticosteroids, and antithyroid drugs. This case uniquely demonstrates that non-thyroid surgery can precipitate thyroid storm without classic hyperthermia, highlighting the need for standardized monitoring protocols in hyperthyroid surgical patients.

## Introduction

Thyroid storm is a severe form of thyrotoxicosis, where a sudden spike in thyroid hormones accelerates metabolism and disrupts multiple organ systems. It is often triggered by stressors like surgery or infections, overwhelming the body's ability to cope. Moreover, thyroid storm can increase the body's sensitivity to catecholamines, exacerbating the hypermetabolic state and leading to organ dysfunction. Some patients exhibit clinical symptoms like high fever, excessive sweating, and rapid heartbeat, linked to heightened catecholamine activity. Globally, the incidence of thyroid crisis among hospitalized patients is approximately 0.2–5.6 per 100,000 per year (1, 2). Individuals diagnosed with hyperthyroidism, whether undergoing thyroid or non-thyroid surgical procedures, are susceptible to thyroid storm during the perioperative period. Reports of intraoperative and postoperative thyroid storm in non-thyroid surgeries are scarce. This rarity is primarily attributed to the prevalent use of antithyroid medications to achieve a euthyroid state preoperatively. Thyroid storm can progress to heart failure, atrial fibrillation, and liver dysfunction (3). If not promptly diagnosed and treated, the mortality rate of thyroid storm can be as high as 8%–25% (4).

In this case study, the patient presented with a confirmed preoperative diagnosis of hyperthyroidism and subsequently exhibited prodromal symptoms indicative of a thyroid storm during the postoperative recovery period following right femoral fixation surgery, manifesting as delayed emergence from anesthesia (≥60 minutes) with tachycardia and unresponsiveness The condition was promptly identified, and emergency management protocols were initiated. Furthermore, a comprehensive review of the literature pertaining to the diagnosis and management of perioperative thyroid storm was conducted. We also reviewed existing literature to highlight diagnostic and management principles applicable to similar cases.

## Case presentation

### Preoperative assessment

A 53-year-old male of Chinese descent was admitted to the orthopedics department for evaluation and management of right thigh pain and deformity following a fall. The patient has a medical history significant for hyperthyroidism and hypertension, both persisting for over two decades. However, he has not adhered to a regular regimen of antithyroid medication and has recently discontinued such treatment. His hypertension is currently managed with oral amlodipine besylate. The patient has a body weight of 90 kg and a height of 172 cm, with a recorded blood pressure of 161/101 mmHg. An electrocardiogram (ECG) indicated sinus tachycardia with a heart rate of 103 beats per minute (bpm).

Between the second and fifth days post-admission, a series of comprehensive laboratory tests and examinations were conducted. The thyroid ultrasound identified a hypoechoic nodule in the left thyroid lobe (C-TIRADS 4a), coarse calcifications in the same lobe (C-TIRADS 4a), cystic nodules in both thyroid lobes (C-TIRADS 2), and heterogeneous changes in the thyroid. The 24-hour Holter monitor revealed sinus tachycardia, with an average heart rate of 111 bpm, a peak of 135 bpm, and a minimum of 85 bpm. Anti-TSHR levels were measured at 1.32 IU/L, within the normal range of 0–1.75 U/L. thyroid function tests indicated the following: free triiodothyronine (FT3) was measured at 6.87 pmol/L (reference range: 3.10–6.80 pmol/L), free thyroxine (FT4) at 21.99 pmol/L (reference range: 12–22 pmol/L), and thyroid-stimulating hormone (TSH) was less than 0.01 mIU/L (reference range: 0.27–4.2 mIU/L). Anti-thyroid peroxidase (Anti-TPO) antibodies were 11.06 IU/ml (reference range: 0–35 IU/ml), and anti-thyroglobulin (Anti-TG) antibodies were 17.74 IU/ml (reference range: 0–120 IU/ml). Additional blood tests did not reveal any significant abnormalities. A computed tomography (CT) scan of the right femur demonstrated a fracture with displacement in the mid-to-upper section. Echocardiography, vascular ultrasound of the lower extremities, and chest CT scan did not show any significant abnormalities.

Notably, the preoperative FT3 elevation (6.87 pmol/L) was mild, which may have contributed to underestimation of storm risk. This underscores that even subclinical hyperthyroidism can precipitate crisis under surgical stress. The levels of Anti-TSHR, Anti-TPO, and Anti-TG were within normal limits, effectively excluding a diagnosis of Graves’ disease and suggesting the absence of significant inflammatory destruction of thyroid tissue at this juncture.

The endocrinologist provided consultations both prior to and following the availability of thyroid function test results. During the second consultation on the fifth day post-admission, it was recommended to initiate bisoprolol at a dosage of 2.5 mg orally once daily to manage heart rate. Concurrently, the methimazole dosage was adjusted from the initial 10 mg daily, as prescribed during the first consultation on the second day post-admission, to 5 mg daily. Additionally, following a cardiology consultation, irbesartan at a dosage of 150 mg orally once daily was prescribed for blood pressure management. The preoperative diagnosis includes a comminuted fracture of the mid-to-upper section of the right femur, hyperthyroidism, and hypertension.

### Intraoperative course

On the sixth day following admission, the patient underwent a closed reduction and intramedullary nailing procedure for a fracture of the right femoral shaft, performed under general anesthesia. On the day of the surgical intervention, the patient adhered to their antihypertensive medication regimen but omitted the administration of antithyroid drugs. Upon entry into the operating theater, the patient's vital signs were recorded as follows: heart rate at 120 beats per minute, blood pressure at 137/72 mmHg, oxygen saturation (SpO_2_) at 92%, respiratory rate at 18 breaths per minute, and body temperature at 36.5°C. The induction of anesthesia was achieved through the administration of cisatracurium, sufentanil, penehyclidine, and etomidate emulsion, followed by the insertion of a 6.5 F endotracheal tube. Anesthetic maintenance was conducted using remifentanil, propofol, and sevoflurane, with dosages titrated as required throughout the surgical procedure. Following the induction of anesthesia, the patient's heart rate gradually decreased to a range of 100–110 bpm, and the systolic blood pressure stabilized within the range of 100–140 mmHg. Ten minutes following the surgical procedure, the patient demonstrated the ability to open his eyes and exhibited an adequate respiratory rate and tidal volume. Subsequent to the administration of neostigmine and atropine, the endotracheal tube was successfully extubated, and the patient was transferred to the post-anesthesia care unit (PACU) for monitoring and recovery.

### Postoperative events

**Figure 1:**
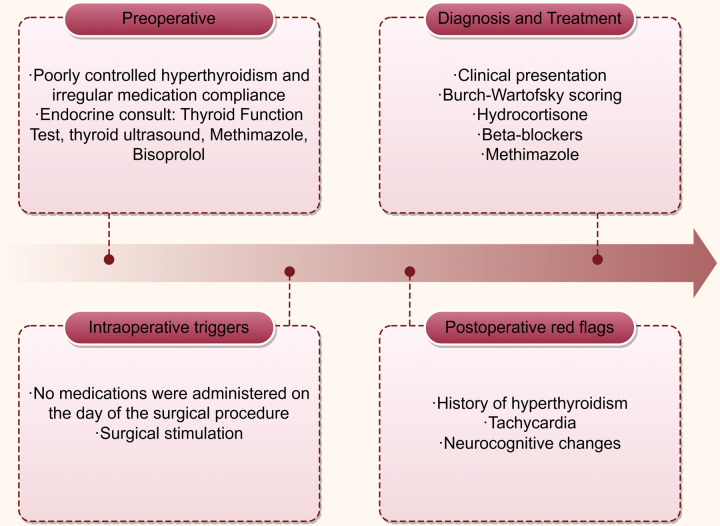
Timeline for diagnosis and treatment of perioperative thyroid crisis development.

Upon arrival in the PACU, the patient's vital signs were as follows: heart rate of 132 beats per minute, respiratory rate of 19 breaths per minute, blood pressure of 125/83 mmHg, SpO_2_ 96%, and body temperature of 36.6°C, with an inhaled oxygen concentration of 30%. Although the patient was able to open his eyes in response to auditory stimuli, he remained unable to follow commands and exhibited a drowsy state. Approximately one hour following admission to the PACU, the patient became unresponsive to verbal stimuli, with a heart rate peaking at 144 bpm, while blood pressure and oxygen saturation levels remained stable. The medical team was invited and reviewed. The patient had a documented history of hyperthyroidism and was assessed using the Burch-Wartofsky Point Scale (Table 1), resulting in a total score of 45 points, with contributions from tachycardia (25 points) and central nervous system disturbance (20 points). This score raised the suspicion of a thyroid storm and fulfilled the Grade 1 criteria as outlined by the Japan Thyroid Association guidelines (Table 2). Due to concerns regarding oral absorption in patients with altered mental status, intravenous administration of hydrocortisone, to provide adrenal support and inhibit T4–T3 conversion, and esmolol, for rapid beta-adrenergic blockade, was prioritized over the immediate use of antithyroid medications. Hydrocortisone was administered intravenously at a dose of 100 mg, and esmolol was delivered via an intravenous micro-pump. The patient's heart rate gradually decreased to 120 bpm, and approximately 20 minutes later, the patient regained full consciousness, exhibiting responsiveness and coherence. The patient did not report any specific discomfort, and after a period of observation without supplemental oxygen, was transferred back to the ward. The patient was advised to resume oral antithyroid medication immediately, and the plan was to add a beta-blocker to manage heart rate. The timeline for the diagnosis and treatment of the patient's perioperative thyroid crisis is shown in Figure 1.

**Table 1:** Burch-Wartofsky point scale.

Clinical features	Score
Thermoregulatory dysfunction (temperature)	
37.2–37.7	5
37.8–38.2	10
38.3–38.8	15
38.9–39.2	20
39.3–39.9	25
≥40.0	30
Central nervous system disturbance	
Absent	0
Mild	10
Moderate (delirium, psychosis, extreme lethargy)	20
Severe (seizures, coma)	30
Gastrointestinal-hepatic dysfunction	
Absent	0
Moderate (diarrhoea, nausea/vomiting, abdominal pain)	10
Severe	20
Tachycardia (beats min^−1^)	
90–109	5
110–119	10
120–129	15
130–139	20
≥140	25
Congestive heart failure	
Absent	0
Mild (pedal oedema)	5
Moderate (bibasilar rales)	10
Severe (pulmonary oedema)	15
Atrial fibrillation	
Absent	0
Present	10
Precipitating event	
Absent	0
Present	10

**Table 2:** The diagnostic criteria for thyroid storm of the Japan thyroid association.

Essential condition	Thyrotoxicosis with elevated levels of free triiodothyronine (FT3) or free thyroxine (FT4)
Clinical features	(1) CNS
	Restlessness, delirium, psychosis, lethargy, coma
	(2) Fever
	Temperature >38°C
	(3) Tachycardia
	Heart rate >130 beats/min or heart rate >130 beats/min in atrial fibrillation
	(4) CHF
	Pulmonary oedema, rales over more than half of the lung field, cardiogenic shock, or Class IV by the New York Heart Association or >Class Ill in the Kilip classification
	(5) Gastrointestinal (Gl) or Hepatic
	Nausea, vomiting, diarrhoea, or a total bilirubin >3.0 mg/dl
Grade of thyroid crisis	1 First combination Thyrotoxicosis and at least one CNS manifestation and fever, tachycardia, CHF, or GI or hepatic manifestations
	1 Alternate combination Thyrotoxicosis and at least 3 combinations of fever, tachycardia, CHF, or GI or hepatic manifestations
	2 First combination Thyrotoxicosis and a combination of 2 of the following: fever, tachycardia, CHF, or GI or hepatic manifestations
	2 Alternate combination Patients who met the diagnosis of thyroid storm Grade 1 except that serum FT3 or FT4 level are not available

On the second postoperative day, the patient's heart rate decreased to 100 bpm. A liver function test yielded the following results: total bilirubin (TBIL) was 19.2 µmol/L (reference range: 5.1–19 µmol/L), direct bilirubin (DBIL) was 10.3 µmol/L (reference range: 1.7–6.8 µmol/L), and indirect bilirubin (IBIL) was 8.9 µmol/L (reference range: 2–21 µmol/L), with a direct to total bilirubin ratio (D/T) of 0.54. On the seventh postoperative day, thyroid function tests indicated that only TSH was abnormal at <0.01 mIU/L (reference range: 0.27–4.2 mIU/L), while all other parameters were within normal limits. The patient was discharged on the tenth postoperative day following substantial healing of the surgical wound. The treatment course and changes in thyroid function over time are presented in Table 3.

**Table 3:** Timeline of treatment course and thyroid function changes.

Time point	Clinical events	Thyroid function tests	Interventions
Preoperative (day 5)	Hypertension, uncontrolled hyperthyroidism	FT3: 6.87 pmol/L (↑), FT4: 21.99 pmol/L (*N*), TSH: <0.01 mIU/L (↓)	Methimazole oral 10 mg/day → 15 mg/day + bisoprolol oral 2.5 mg/day
Day of surgery	Omitted antithyroid drugs; general anesthesia for femoral fixation	Not measured	Anesthesia: remifentanil, propofol, sevoflurane
PACU admission	Delayed emergence (HR: 144 bpm, altered consciousness)	Not measured	Hydrocortisone 100 mg IV + esmolol infusion
Post-op day 2	HR stabilized to 100 bpm; liver dysfunction	TBIL: 19.2 µmol/L (↑), DBIL: 10.3 µmol/L (↑)	Continued methimazole(oral) + beta-blockers(oral)
Post-op day 7	HR 75 bpm, BP 123/76 mmHg	TSH: <0.01 mIU/L (↓), FT3: 5.17 pmol/L (*N*), FT4: 20.18 pmol/L (*N*)	Continued methimazole(oral) + beta-blockers(oral)
Post-op day 10	Clinical recovery; wound healing	Not measured	Continue taking methimazole orally as prescribed after discharge.

## Literature review

We performed a systematic review of the PubMed/Medline database, covering the period from 2005 to 2025, employing the keywords “thyroid crisis” OR “thyroid storm” in conjunction with “perioperative,” “surgery,” “non-thyroid surgery,” or “nonthyroid surgery” using Boolean operators: (thyroid crisis OR thyroid storm) AND (perioperative OR surgery OR non-thyroid surgery). Initially, 211 publications were screened, of which 18 cases satisfied the inclusion criteria for peer-reviewed reports documenting intraoperative or postoperative thyroid storm during non-thyroid surgeries (Table 4). This review was conducted in accordance with the PRISMA guidelines. The surgical procedures included abdominal (*n* = 5), orthopedic (*n* = 4), cardiothoracic (*n* = 4), obstetric (*n* = 3), and other procedures (*n* = 2). In this cohort, thyroid storm was observed intraoperatively in 8 patients and postoperatively in 10 patients. Concerning the correlation with pre-existing thyroid conditions, 5 patients had a documented history of thyroid disorders, while 3 patients had documented thyroid symptoms preoperatively but lacked formal diagnosis, suggesting potential oversight in preoperative assessment. Additionally, 8 patients were unaware of any underlying thyroid conditions before undergoing surgery. The most prevalent clinical manifestations included tachycardia (100%, 18/18), fever (56%, 10/18), central nervous system symptoms (27%, 5/18), and heart failure (16%, 3/18). Diagnosis was primarily reliant on thyroid hormone assays and the Burch-Wartofsky Point Scale, with initial evaluations largely guided by clinical presentation. The majority of patients exhibited favorable responses to treatment with antithyroid medications, corticosteroids, and adjunctive supportive therapies. Notably, overall prognosis was favorable, with only one reported mortality due to a postoperative pulmonary complication.

**Table 4:** Summary of cases with thyroid storm in the perioperative period of nonthyroidal surgery.

Case	Age	Sex	Surgical type	History of thyroid disease(yes/no/not obtained^a^/undefined^b^)	Clinic presentation	Occurrence time	Diagnosis	Treatment	Prognosis
Shao 2024 (16**)**	52	F	Total knee arthroplasty	Not obtained	Tachycardia	Intra-Op	Thyroid function test, radioactive iodine uptake test, Burch and Wartofsky score	Methimazole, β-blockers	Recovery
Chen 2022 (17)	38	F	Cesarean section	Yes	Nausea, abdominal pain, tachycardia, fever, hypertension, disturbance of consciousness, blood oxygen desaturation	Intra-Op	Clinical presentation, Burch and Wartofsky score	Propylthiouracil, hydrocortisone	Recovery
Sungworawongpana 2022 (18)	19	F	Laparoscopic appendectomy	Undefined	Fever, tachycardia	Intra-Op	Thyroid function test, Burch and Wartofsky score	Methimazole, hydrocortisone, propranolol	Recovery
Hu 2022 (19)	11	M	Curettage of right femur, bone grafting and osteotomy and fixation	Yes	Dizziness, nausea, vomiting, fever, tachycardia	Post-Op	Clinical presentation	Propranolol, hydrocortisone, saturated solution of potassium iodine	Recovery
Iwahara 2021 (20)	76	M	Left radical nephrectomy and thrombectomy	Not obtained	Central nervous system disturbance, fever, tachycardia, congestive heart failure, hepatic manifestation	Post-Op	Thyroid function test, clinical presentation	Inorganic iodide	Recovery
John A. 2021 (21)	49	F	Dilation and curettage	Undefined	Nervous system disturbance, fever, tachycardia, congestive heart failure, hepatic manifestation	Post-Op	Thyroid function test, Burch and Wartofsky score, clinical presentation	Propylthiouracil, hydrocortisone, propranolol, iodine solution	Recovery
Lee 2020 (22)	74	F	Coronary artery bypass graft	Yes	TACHYCARDIA	Post-Op	Thyroid function test, Burch and Wartofsky score,	Esmolol, cortisol, antithyroid medication, antibiotic	Died
Yamazaki 2020 (23)	40	M	Mitral annuloplasty	NO	Tachycardia	Post-Op	Thyroid function test, clinical presentation	Lugol's iodine, thiamazole, hydrocortisone	Recovery
Sugisaki 2019 (24)	32	F	Caesarean section	Yes	Fever, tachycardia, heart failure	Post-Op	Burch and Wartofsky Score, clinical presentation, thyroid function test	Thiamazole, potassium iodide	Recovery
Mohammadali 2018 (25)	30	F	Thoracoscopic resection of the mass and total thymectomy	No	Fever, tachycardia, hypercapnic respiratory failure, agitated and tremulous	Post-Op	Burch and Wartofsky Score, clinical presentation, thyroid function test	Propylthiouracil, esmolol, saturated solution of potassium iodide (sski), hydrocortisone	Recovery
Pride 2018 (26)	46	M	Esophagogastroduodenoscopy	Undefined	Tachycardia, hypertension	Intra-Op	Burch and Wartofsky score, clinical presentation, thyroid function test	Unclear	Recovery
Emi 2018 (27)	43	F	Intracranial surgery	Undefined	Disturbed consciousness, hypertension, tachycardia	Post-Op	Clinical presentation, thyroid function test	Unclear	Recovery
Zhang 2018 (28)	32	F	Huge pelvic mass resection	Yes	Tachycardia	Intra-Op	Clinical presentation, thyroid function test	Neostigmine, propylthiouracil, hydrocortisone	Recovery
Huzurbazar 2014 (29)	21	F	Cervical spine surgery	Not obtained	Restlessness, hypotension, tachycardia, fever, dyspnea, blood oxygen desaturation	Post-Op	Clinical presentation, thyroid function test, Burch and Wartofsky score	Corticosteroids, neomercazole	Recovery
Bish 2010 (30)	45	F	Coronary artery bypass grafting	Undefined	Tachycardia, fever, jaundice, depressed mental status	Post-Op	Clinical presentation, thyroid function test, Burch and Wartofsky score	Potassium iodide, methimazole, hydrocortisone	Recovery
Tamada 2010 (31)	35	M	Fracture surgery	Undefined	Tachycardia	Intra-Op	Thyroid function test, clinical presentation	Landiolol	Recovery
Kathleen 2008 (32)	18	M	Pituitary tumor surgery	Undefined	Tachycardia	Intra-Op	Thyroid function test, clinical presentation	Propylthiouracil, beta-adrenergic blocking agents	Recovery
Nakamura 2006 (33)	30	M	Left tympanoplasty	Undefined	Tachycardia, fever	Intra-Op	Thyroid function test, clinical presentation	Thiamazole, propranorol	Recovery

Intra-Op, intraoperative; Post-Op, postoperative.

^a^ Not obtained: The patient exhibited thyroid-related clinical symptoms preoperatively, but the medical history was not obtained by the physician.

^b^ Undefined: The patients’ thyroid condition was undiagnosed prior to surgery but was identified postoperatively.

## Discussion

This case illustrates the diagnostic complexity of thyroid storm in postoperative settings, where anesthesia-related confounders (e.g., delayed emergence, hemodynamic fluctuations) may mimic or mask classic features. Unlike typical presentations with hyperthermia and overt hypermetabolism (1), our patient exhibited only tachycardia and altered consciousness, underscoring the need for heightened vigilance in non-thyroid surgeries. Crucially, our patient did not have a fever and had normal FT4 levels, making diagnosis under anesthesia challenging. The patient's anti-thyrotropin receptor antibody (Anti-TSHR) level was 1.32 IU/L, within the normal range (0–1.75 IU/L), reducing the likelihood of Graves’ disease. However, thyroid ultrasound revealed hypoechoic nodules classified as C-TIRADS 4a, suggesting toxic nodular goiter as the probable underlying cause of hyperthyroidism.

Numerous precipitating factors can lead to a perioperative thyroid storm, with its primary clinical manifestations exhibiting considerable variability. These manifestations may impact the central nervous system, cardiovascular system, digestive system, and thermoregulatory processes, as outlined in Table 5 (5). During episodes of acute stress, there is an enhanced cellular response to thyroid hormones. An increase or abrupt release of free hormones results in an amplified response to catecholamines. A rapid increase in thyroid hormone levels, rather than their absolute amounts, is believed to trigger a thyroid storm. Although a trigger is usually needed for a thyroid storm in hyperthyroidism and thyrotoxicosis, up to 30% of cases occur without an identifiable cause.

**Table 5:** Precipitating factors and clinical manifestations of thyroid storm.

Patient condition	Inducing factor	Clinical feature
Waking state	Trauma, infection, pulmonary embolism, myocardial infarction, pregnancy-related disorders, diabetic ketoacidosis, thyroid or non-thyroid surgery,sudden discontinuation of antithyroid medication,excessive iodine intake	Central nervous system: irritability, delirium, coma, seizures Cardiovascular system: tachycardia, arrhythmia, heart failure, hypotension digestive System: nausea, vomiting, abdominal pain, jaundice Temperature regulation: fever, dehydration
Anesthesia state		Central nervous system: seizures, delayed awakening Cardiovascular system: tachycardia, arrhythmia Temperature regulation: fever or afebrile

Thyroid storm diagnosis primarily depends on clinical criteria, with tools like the Burch-Wartofsky Point Scale and Japan Thyroid Association guidelines commonly employed. These methods, however, have limitations, including reduced sensitivity for mild cases and challenges in differentiating thyroid storm from other critical conditions. Additionally, reliance on subjective clinical judgment can lead to variability among healthcare professionals (6). Predicting thyroid storm involves assessing clinical symptoms alongside the Burch-Wartofsky Point Scale, as detailed in Table 1 (3). A score of 45 or greater is highly suggestive of thyroid storm, a score of 25–44 is suggestive of impending storm, and a score below 25 is unlikely to represent thyroid storm. Key clinical signs include hyperthermia, tachycardia, central nervous system disturbances, and gastrointestinal or hepatic dysfunction. Patients may exhibit symptoms such as high fever (temperature >39°C), tachycardia (heart rate >140 bpm), altered consciousness, diarrhea, jaundice, and abnormal thyroid function tests (e.g., decreased TSH, elevated free T3 and T4) (1).Nonetheless, atypical presentations complicate the diagnostic process, as certain patients may initially exhibit acute abdominal symptoms, resulting in potential misdiagnosis. Concurrently, others may experience a thyroid storm alongside other conditions, such as subacute thyroiditis or subarachnoid hemorrhage, further complicating accurate diagnosis (7, 8). Additionally, under anesthesia, some clinical indicators from the Burch-Wartofsky Point Scale may be obscured. In response to these challenges, the Japan Thyroid Association introduced diagnostic criteria in 2012, as detailed in Table 2 (9). Grade 1 indicates a definite thyroid storm and Grade 2 a suspected thyroid storm. Cases are excluded if other underlying diseases clearly cause any of the following symptoms: fever (e.g., pneumonia and malignant hyperthermia), impaired consciousness (e.g., psychiatric disorders and cerebrovascular disease), heart failure (e.g., acute myocardial infarction), and liver disorders (e.g., viral hepatitis and acute liver failure). Therefore, it is difficult to determine whether the symptom is caused by a thyroid storm or is simply a manifestation of an underlying disease; the symptom should be regarded as being due to a thyroid storm that is caused by these precipitating factors (34). Consequently, it is advisable to employ both scoring systems when evaluating patients suspected of experiencing a thyroid storm to enhance diagnostic precision.

Delayed emergence from anesthesia is a multifaceted issue attributable to various factors. It is primarily attributed to the residual effects of anesthetic and analgesic agents, whose metabolism and clearance rates vary among individuals, leading to prolonged awakening times (10). Additionally, less common causes such as cerebral edema or unexpected cerebral venous sinus thrombosis, which may not be immediately evident postoperatively, can significantly impede recovery (11). Neurocognitive recovery delays are particularly concerning in elderly patients and may be associated with the type of anesthetic used, pre-existing conditions like diabetes or prior cerebrovascular events, and intraoperative factors such as hypothermia (12). Moreover, mental health status plays a role, with evidence indicating that patients with pre-existing depression are more susceptible to delayed recovery, potentially due to depression's negative impact on physiological recuperation (13). Thus, delayed emergence from anesthesia results from an interplay of pharmacological, physiological, and psychological factors.

In our case, the potential causes of delayed emergence from anesthesia, such as residual anesthetic agents or metabolic disturbances (including hypoglycemia and electrolyte imbalances), were considered. However, an arterial blood gas analysis was not performed in the post-anesthesia care unit (PACU), representing a significant oversight. Future protocols ought to integrate arterial blood gas (ABG) analysis to facilitate a comprehensive evaluation of delayed emergence and metabolic disturbances, particularly in patients who demonstrate abrupt alterations in consciousness following surgery. This omission hindered the exclusion of carbon dioxide accumulation, electrolyte imbalances, or other potential causes for the delayed emergence. Nevertheless, based on the patient's symptoms at the time, the Burch-Wartofsky Point Scale was calculated as follows: tachycardia (144 beats/min, 25 points), altered mental status (20 points), and absence of fever (0 points), resulting in a total score of 45 points, which strongly indicates a thyroid storm. According to the criteria set by the Japan Thyroid Association, the presence of thyrotoxicosis accompanied by central nervous system disturbance and tachycardia qualified the patient for a grade 1 diagnosis. As a result, a thyroid storm was suspected, and treatment with hydrocortisone and esmolol was given following American and Japan Thyroid Association guidelines (9, 14). Due to the altered mental status and concerns about oral absorption, methimazole was not administered immediately postoperatively. The patient's rapid response to hydrocortisone and esmolol, and subsequent quick awakening, further confirmed the diagnosis of thyroid storm.

Elective surgeries should be postponed for hyperthyroid patients showing symptoms like tachycardia, altered consciousness, fever, and gastrointestinal issues, which indicate a higher risk of thyroid storm. The attainment of a euthyroid state may require a duration ranging from 6 weeks to 18 months and should be managed under the supervision of an endocrinologist (9, 14). The most effective preoperative prophylactic treatment for hyperthyroid patients preparing for surgery involves a comprehensive approach: reducing thyroid hormone synthesis and secretion through the use of thionamides, iodine solutions, or both; safeguarding the cardiovascular system and maintaining hemodynamic stability with beta-blockers; and decreasing circulating free T3 levels while substituting cortisol in patients with adrenal insufficiency through the administration of corticosteroids. In a systematic review on the risk of perioperative thyroid storm conducted by Nikki et al. (4), it was observed that thyroid storms may occur intraoperatively or postoperatively, irrespective of the use of single-drug or combination therapies in the preoperative phase. Notably, this risk is present in both euthyroid and hyperthyroid patients. When selecting an anesthetic strategy, regional anesthesia and peripheral nerve blocks are preferred over general anesthesia whenever feasible. In cases where regional anesthesia techniques are not applicable to the surgical procedure, general anesthesia should be administered with strategies to mitigate intubation responses, ensure adequate analgesia and deep anesthesia, and facilitate smooth extubation (15).

The patient's incomplete adherence to methimazole reflect a critical gap in perioperative optimization. Current guidelines emphasize achieving euthyroidism before elective surgery (9, 14), yet this case demonstrates how subclinical hyperthyroidism (evidenced by normal FT4 but suppressed TSH) can still precipitate storm under surgical stress. Notably, the omission of antithyroid drugs on the day of surgery may have exacerbated hormone release, highlighting the need for strict medication compliance protocols.

According to the treatment guidelines established by the American and the Japan Thyroid Association (9, 14), the management protocol for thyroid storm during and post-surgery encompasses several key interventions. Seizure-like symptoms are treated with diazepam (0.15–0.2 mg/kg, max 10 mg) or propofol (1–2 mg/kg). Fever should be managed with physical cooling methods, avoiding NSAIDs due to their risk of increasing free thyroid hormone release. To inhibit thyroid hormone synthesis, oral propylthiouracil (PTU) is given with a loading dose of 500–1,000 mg, then 200–250 mg every 4 hours, while methimazole can be used orally (60 mg) or intravenously (30 mg daily). One hour after thionamides, iodine agents like Lugol's solution or iopanoic acid are administered to block hormone release. Beta-blockade is achieved with intravenous esmolol (1 mg/kg bolus, then 150 mcg/kg/min infusion) to maintain heart rate under 130 bpm; propranolol or metoprolol are alternatives. To inhibit peripheral T4 to T3 conversion, hydrocortisone (300 mg loading, then 100 mg IV every 8 hours) or dexamethasone (8 mg IV daily) is used. For atrial fibrillation, digoxin is dosed at a total loading of 8–12 mcg/kg titrated over 12 hours, and amiodarone is given if left ventricular dysfunction is present (150 mg over 10 minutes, then 1 mg/min for 6 hours, followed by 0.5 mg/min, max 2.4 g/day). Acute heart failure is managed with IV furosemide (0.5–1 mg/kg), and cardiogenic shock may require dobutamine (2.5–10 mcg/kg/min) and norepinephrine (0.03–0.3 mcg/kg/min) infusions.

While thyroid storm is well-documented in thyroidectomies (4), its occurrence after orthopedic surgery is rare. This case suggests that even minor surgical trauma can trigger storm in poorly controlled hyperthyroidism, particularly when cardiovascular stressors (e.g., pain, fluid shifts) are present. We recommend the implementation of mandatory preoperative endocrine consultations for all patients with hyperthyroidism who are scheduled to undergo non-thyroid surgical procedures, irrespective of their biochemical stability. Such consultations could potentially prevent the omission of necessary medications and ensure optimal thyroid function prior to surgery.

## Conclusion

This case highlights the crucial impact of perioperative medication adherence—omission of antithyroid drugs on the day of surgery likely precipitated the storm. Notably, the absence of hyperthermia highlights the risk of missed or delayed diagnosis when relying solely on classic signs. Our experience underscores the need for heightened vigilance and suggests that monitoring protocols for hyperthyroid patients undergoing any surgery should be standardized and multidisciplinary.

Key lessons from this case emphasize the importance of comprehensive perioperative management in patients with hyperthyroidism. Achieving a euthyroid state with antithyroid drugs and beta-blockers before surgery is crucial to prevent thyroid storm, with input from endocrinology being essential. During and after surgery, careful monitoring is necessary as these patients are still at risk, even in non-thyroid procedures. Anesthetic techniques should aim to minimize sympathetic stimulation and hemodynamic instability. Quick diagnosis using the Burch-Wartofsky Scale and Japan Thyroid Association criteria, along with immediate treatment with beta-blockers, corticosteroids, and antithyroid medications, is critical for survival. Educating patients on medication adherence and early symptom detection is also key to avoiding recurrence.

This case adds to the limited literature on thyroid storm triggered by non-thyroid surgeries and reinforces the need for standardized perioperative protocols. Future research should focus on developing risk stratification tools and evaluating anesthetic strategies tailored for hyperthyroid patients across various surgical settings.

## Data Availability

The original contributions presented in the study are included in the article/Supplementary Material, further inquiries can be directed to the corresponding author.
